# Be Constantly Employed

**Published:** 1855-06

**Authors:** 


For the Journal of Health.

### BE CONSTANTLY EMPLOYED.

We believe it is universally observed, by such as have looked upon life with a thoughtful eye, that those whom necessity requires to be constantly employed, are the most healthy and cheerful among mankind. A constant employment of time, is therefore conducive to health and happiness. It has been wisely ordered by the Ruler of the Universe, that man should obtain a livelihood by the sweat of his brow, and that this very labor should give health to his body, and contentment to his mind. Nature, by her mysterious promptings, teaches us all that exercise is necessary; for unless all the muscles are daily called into use, they will soon decrease in size and power. So without proper exercise, our mortal frame will soon be shorn of its strength. By being constantly employed, it helps to while away many an hour which would otherwise have been spent in thinking of the unhappy past. By being engaged in some pleasant employment, your sadness has no time to fasten on your spirits. Employment prolongs life. It is true it cannot subdue death; but it can defer that hour, and cause many enjoyments to linger around our pathway, and make it a pleasure to live. We would earnestly request you, dear reader, to employ a portion of your time in worshipping God. You will then pass your days with comfort to yourself and those around you, and when the ties which bind you to earth are dissevered, you will receive the welcome plaudit, "Come ye blessed of my Father, inherit the kingdom prepared for you."

